# Prediction model for elevated intraocular pressure risk after silicone oil filling based on clinical features

**DOI:** 10.3389/fmed.2023.1340198

**Published:** 2024-01-09

**Authors:** Wen Fan, Chaohe Zhang, Lexin Ge, Na Su, Jiaqin Chen, Siyao Song, Yasha Wang, Songtao Yuan

**Affiliations:** ^1^Department of Ophthalmology, The First Affiliated Hospital of Nanjing Medical University, Nanjing, China; ^2^Key Laboratory of High Confidence Software Technologies, Ministry of Education, Peking University, Beijing, China; ^3^School of Computer Science, Peking University, Beijing, China; ^4^National Engineering Research Center of Software Engineering, Peking University, Beijing, China; ^5^Software College, Northeastern University, Shenyang, China

**Keywords:** prediction model, intraocular pressure, ocular hypertension, pars plana vitrectomy, silicone oil, biological parameter

## Abstract

**Background:**

To evaluate risk factors and further develop prediction models for intraocular pressure elevation (IOP) after vitreoretinal surgery with silicone oil tamponade to support clinical management.

**Methods:**

A retrospective study analyzed 1,061 eyes of 1,061 consecutive patients that presented to the Jiangsu Province Hospital between December 2015 and December 2020, the IOP was measured from the preoperative visit and at the 1-week, 1-month, 3-month, and 6-month visits, and the final postoperative visit before silicone oil removal. Four machine learning methods were used to carried out the prediction of IOP elevation: Decision Tree, Logistic Regression, Random Forest, and Gradient-Boosted Decision Trees (GBDT) based on features including demographic and clinical characteristics, preoperative factors and surgical factors. Predictors were selected based on the *p*-value of the univariate analysis.

**Results:**

Elevated intraocular pressure developed in 26.01% of the eyes postoperatively. Elevated intraocular pressure primarily occurred within 1–2 weeks after surgery. Additionally, the majority of IOP values were distributed around 25–40 mmHg. GBDT utilizing features with *p*-values less than 0.5 from the hypothesis testing demonstrated the best predictive performance for 0.7944 in accuracy. The analysis revealed that age, sex, hypertension, diabetes, myopia, retinal detachment, lens status and biological parameters have predictive value.

**Conclusion:**

Age, sex, hypertension, diabetes, myopia, retinal detachment, lens status and biological parameters have influence on postoperative intraocular pressure elevation for patients with silicone oil tamponade after pars plana vitrectomy. The prediction model showed promising accuracy for the occurrence of IOP elevation. This may have some reference significance for reducing the incidence of high intraocular pressure after pars plana vitrectomy combined with silicone oil filling.

## 1 Introduction

Silicone oil (SO) has become an essential tamponade agent in vitreoretinal surgery due to its high surface tension since its introduction by Cibis in 1962 (1). However, despite its successful use by retina surgeons for several decades, complications associated with silicone oil endotamponade have been reported. These include increased intraocular pressure (IOP), ocular hypotony, cataract formation in phakic eyes, band keratopathy in corneas, and silicone oil emulsification (2, 3). Elevated IOP is particularly prevalent and severe among these complications (4). The incidence of elevated IOP varies from 2.2 to 56.0% related to SO endotamponade in different studies (5–8). Discrepancies in the definitions of ocular hypertension, postoperative time points, and retinal diseases contribute to this variation. Additionally, each study incorporates different inclusion and exclusion criteria. The underlying pathological mechanisms of IOP elevation are multifactorial and remain uncertain. They may include overfilling of silicone oil, anterior chamber inflammatory activity, pupillary block, anterior migration of SO, and preexisting glaucoma (9–12). In public hospitals, the limited use of inert gas is attributed to policy constraints. Consequently, silicone oil filling is the predominant choice for patients undergoing vitrectomy combined with intraocular filling surgery. As a result, there is a considerable prevalence of silicone oil-filled eyes in China, necessitating an analysis of the risk factors associated with elevated intraocular pressure following silicone oil filling.

Since the occurrence of postoperative IOP elevation is the result of multiple factors, conventional single-factor analysis fails to provide a comprehensive analysis, and there is no consensus regarding the risk factors derived from current study analyses. With the advancement of data science, machine learning models have gained popularity in predictive tasks across various domains, including healthcare. However, the medical field places a high premium on model interpretability, leading to the development of interpretable predictive models for clinical analysis. In this study (2, 13), we presented a predictive model for further analysis, offering clinicians not only predicted values for assistance but also interpretability to discern the importance of different features and provide more precise clinical reference.

## 2 Materials and methods

This study was approved by the ethics of committees of The First Affiliated Hospital of Nanjing Medical University (Jiangsu Province Hospital) and conducted in accordance with the tenets of the Declaration of Helsinki (2022-SR-200). As this is a retrospective study with data processed anonymously, informed consent was waived.

This retrospective study enrolled 1,061 eyes of 1,061 consecutive patients that presented to the Jiangsu Province Hospital between December 2015 and December 2020. These cases were confirmed by experienced researchers. All eyes had a vitreoretinal condition treated by pars plana vitrectomy (PPV) with SO endotamponade. Eyes with corneal pathology, a previous history of glaucoma or preoperative ocular hypertension (IOP > 21 mmHg), or patients with severe data deficiencies were excluded. Demographic data, a detailed ocular and systemic history, and the etiology of the PPV were recorded for each patient pre-operatively.

Vitreoretinal surgery was indicated for various conditions, including retinal detachment (RD), macular hole (MH), choroidal detachment, vitreous hemorrhage (VH), and diabetic retinopathy (DR). All procedures were performed under either local or general anesthesia. PPV, air/fluid exchange, and SO were employed in all patients. Following surgery, the eyes were maintained at a slightly hypotonic state, and intraocular pressure was assessed through manual palpation.

Patient age, sex, lens status, presence of diabetes, hypertension, biological parameters, surgical indication and surgical factors were recorded. Biological parameters were measured by ZEISS IOL Master500 before surgery. The IOP measured by Canon TX-20 automatic non-contact tonometer were obtained for both the operative and fellow eyes from the preoperative visit and at the 1-week, 1-month, 3-month, 6-month visits, and at the final postoperative visit. The follow-up ended after silicone oil removal. Silicone Oil was removed about 3–6 months after implantation. IOP less than 21 mmHg was predefined as normal. If the intraocular pressure exceeds 21 mmHg at any point during the follow-up process, it is diagnosed as elevated intraocular pressure, and the appropriate treatment method is selected based on the specific numerical value. Commonly used IOP-lowering drugs include Brinzolamide and Timolol Maleate Eye Drops, Carteolol Hydrochloride Eye Drops, Brimonidine Tartrate Eye Drops and Travoprost Eye Drops. For patients whose IOP was not effectively controlled with IOP-lowering medications, we performed further operations or surgeries to control IOP, such as paracentesis of anterior chamber, trabeculectomy and drainage valve implantation.

Data were analyzed using Python 3.6.9 with package: SciPy (1.2.1), Statsmodels (0.11.1), NumPy (1.19.4) and scikit-learn (0.24.1). Continuous variables were expressed as means (standard deviations), and were compared with the Mann–Whitney U test. Categorical variables were expressed as number (%) and compared by χ2 test. A *p*-value of less than 0.05 was considered a statistically significant difference. Four machine learning methods were used to carried out the prediction of IOP elevation: Decision Tree, Logistic Regression, Random Forest, and Gradient-Boosted Decision Trees (GBDT). Among these, GBDT achieved the best predictive performance. The selection of predictive features was based on the *p*-value from the above hypothesis testing.

## 3 Results

### 3.1 Demographic and clinical characteristics

**Figure 1:**
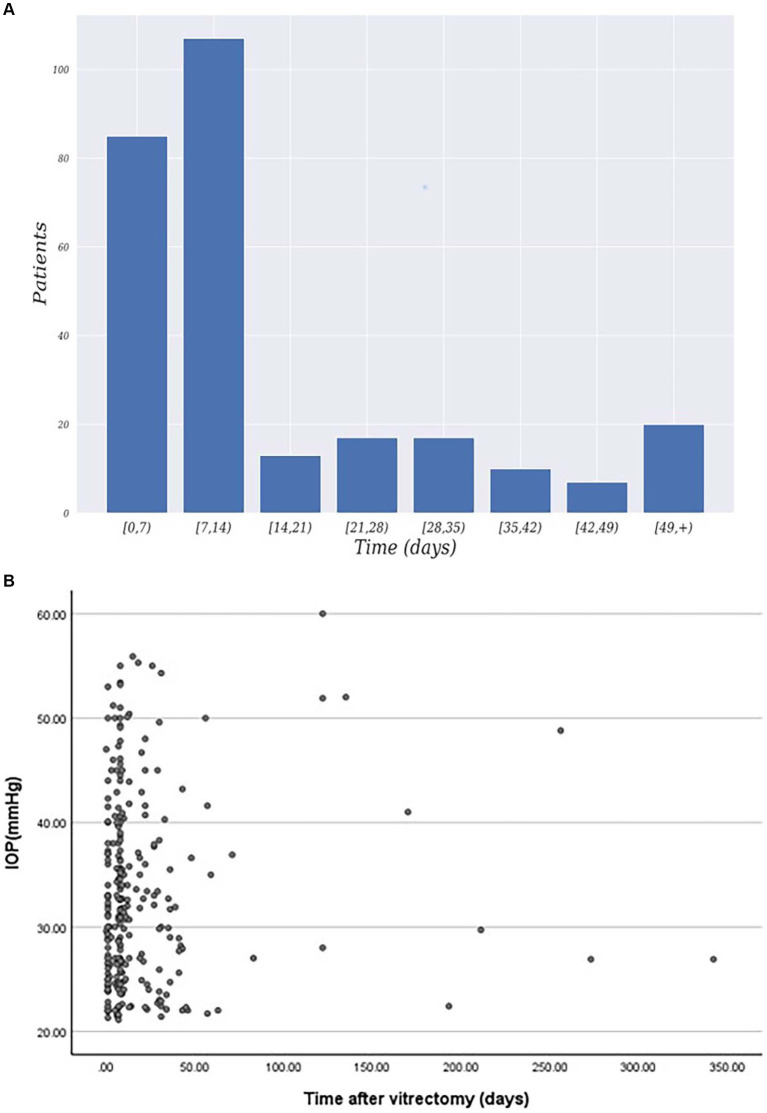
**(A)** Distribution of patients exhibiting elevated intraocular pressure time after surgery. **(B)** Time and IOP values for postoperative intraocular pressure elevation.

The baseline characteristics of the study cohort were presented in Table 1. The study included 1,061 patients who underwent pars plana vitrectomy, with a mean age of 58.52 years ±12.81 and range of 11 to 93 years. Of the total number of patients, 563 (53.06%) were male, and 538 (46.94%) were female. Diabetes mellitus was present in 205 patients (19.32%), systemic hypertension in 301 patients (28.37%), and myopia in 247 patients (23.28%). The primary indication for surgery in the study eye was retinal detachment in 771 patients (72.67%), followed by vitreous hemorrhage in 171 patients (16.12%) and macular hole in 122 patients (11.50%). Among the 276 patients with IOP elevation, Figure 1 illustrated that elevated intraocular pressure primarily occurs within 1–2 weeks after surgery. Additionally, the majority of IOP values are distributed around 25–40 mmHg.

**Table 1:** The baseline characteristics of the study cohort.

Characteristics	Value
Male	563 (53.06%)
Age at surgery, years	58.52 ± 12.81 (11–93)
A history of diabetes mellitus	205 (19.32%)
A history of high blood pressure	301 (28.37%)
Myopia	247 (23. 28%)
Retinal detachment	771 (72.67%)
Macular hole	122 (11.50%)
Choroidal detachment	61 (5.75%)
Vitreous hemorrhage	171 (16.12%)

### 3.2 Preoperative risk factors

The analysis of preoperative risk factors for intraocular pressure elevation is presented in Table 2. Out of the 1,061 patients included in the study, 276 (26.01%) developed postoperative IOP elevation, 785 (73.99%) showed normal postoperative IOP. We compared various variables among all the patients and found that age at surgery, sex, myopia, retinal detachment, vitreous hemorrhage, hypertension and diabetes were associated with a higher risk of increased IOP. No significant associations were observed between other characteristics and the development of elevated IOP. Specifically, the age of patients with IOP elevation was significantly lower compared to those with normal IOP (*p* < 0.001, OR = 0.96 [0.95–0.97]). Gender also showed a significant difference (*p* = 0.02, OR = 1.39 [1.05–1.83]). Among patients with IOP elevation, the underlying ocular diseases necessitating pars plana vitrectomy (PPV) included retinal detachment, macular hole, choroidal detachment, vitreous hemorrhage, and diabetic retinopathy. Patients with retinal detachment (*p* < 0.001, OR = 6.71 [4.16–10.83]), diabetes (*p* = 0.045, OR = 0.69 [0.47–0.99]), hypertension (*p* = 0.018, OR = 0.68 [0.49–0.94]), vitreous hemorrhage (*p* = 0.002, OR = 0.51 [0.33–0.78]), and myopia (*p* < 0.001, OR = 1.84 [1.36–2.51]) were more susceptible to developing increased IOP. On the other hand, the presence of macular hole and choroidal detachment did not appear to influence the risk of elevated IOP.

**Table 2:** The preoperative risk factors for intraocular pressure elevation.

	Eyes		OR	95% CI	*p*-value
	With IOP > 21 mmHg (*n* = 276)	With IOP ≤ 21 mmHg (*n* = 785)			
**Preoperative characteristics**					
Age, years	53.34 ± 13.38	60.34 ± 12.08	0.96	0.95–0.97	<0.001
Sex					
Male	163	400	1.39	1.05–1.83	0.020
Female	113	385			
Diabetes					
Yes	42	163	0.69	0.47–0.99	0.045
No	234	622			
Hypertension					
Yes	63	238	0.68	0.49–0.94	0.018
No	213	547			
Myopia					
Yes	88	159	1.84	1.36–2.51	<0.001
No	188	626			
Retinal detachment					
Yes	256	515	6.71	4.16–10.83	<0.001
No	20	270			
Macular hole					
Yes	28	94	0.83	0.53–1.30	0.412
No	248	691			
Choroidal detachment					
Yes	21	40	1.53	0.89–2.65	0.123
No	255	745			
Vitreous hemorrhage					
Yes	28	143	0.51	0.33–0.78	0.002
No	248	642			

### 3.3 Surgical factors

Effects of surgical factors are shown in Table 3. Among all 1,061 patients, 891 patients were with phakic, 22 patients were aphakic, 148 patients were Pseudophakic. Lens status had significant statistical differences. Besides, other continuous variables were also analyzed, such as axial length (AL), anterior chamber depth (ACD), lens thickness (LT), central corneal thickness (CCT), white-to-white value (WTW). Eyes with longer axial length, deeper ACD and longer WTW were significantly associated with elevated IOP. Patients with elevated IOP had a mean AL of 24.82 ± 3.77, which was significantly longer than patients with normal IOP (23.88 ± 2.76) (*p* < 0.001, OR = 1.10 [1.05–1.16]). Similar findings were observed for ACD (*p*<0.001, OR = 1.83 [1.35–2.49]), and WTW (*p* = 0.04, OR = 1.34 [0.99–1.80]). However, no significant difference was found in CCT and LT between the two groups. A total of 36 (3.4%) patients had 1000cSt silicone oil implanted, while the remaining 1,025 (96.6%) patients received 5000cSt silicone oil. SO viscosity was not found to be associated with the development of increased IOP (*p* = 0.799). Meanwhile, pars plana vitrectomy combined cataract extraction conferred a reduced risk for increased IOP (*p* < 0.001, OR = 0.44 [0.33–0.58]).

**Table 3:** The surgical factors for intraocular pressure elevation.

	Eyes		OR	95% CI	*p*-value
	With IOP > 21 mmHg (*n* = 276)	With IOP ≤ 21 mmHg (*n* = 785)			
**Intraoperative characteristics**					
Lens status					
Phakic	213	678	0.53	0.38–0.76	<0.001
Aphakic	13	9	4.80	1.97–11.71	
Pseudophakic	50	98	1.55	1.07–2.25	
Biological parameter					
Axial length	24.82 ± 3.77	23.88 ± 2.76	1.10	1.05–1.16	<0.001
Anterior chamber depth	3.25 ± 0.55	3.09 ± 0.48	1.83	1.35–2.49	<0.001
Lens thickness	4.82 ± 0.75	4.85 ± 0.66	0.95	0.71–1.26	0.509
Central corneal thickness	543.89 ± 54.53	540.16 ± 38.28	1.00	1.00–1.01	0.074
WTW	11.59 ± 0.56	11.51 ± 0.53	1.34	0.99–1.80	0.041
Viscosity of silicone oil					
1,000 cSt	7	29	0.61	0.25–1.48	0.799
5,000 cSt	269	756	1.19	0.44–3.26	
DK-line injection					
Yes	208	605	0.93	0.67–1.30	0.669
No	68	180			
Combined cataract extraction					
Yes	136	542	0.44	0.33–0.58	<0.001
No	140	243			

### 3.4 Prediction models

Four machine learning methods:

- Decision tree: a decision tree is a flowchart-like model that makes decisions based on a series of questions about the data. In our study, it was used to predict IOP elevation by asking a series of intuitive, yes-or-no questions based on patient characteristics.
- Logistic regression: logistic regression is a statistical model that estimates the probability of a binary outcome (such as IOP elevation) based predictor variables. In our context, it assessed the likelihood of IOP elevation by analyzing patient characteristics.
- Random forest: a random forest is an ensemble learning method, which operates by constructing multiple decision trees during training and outputting the class that is the mode of the classes of the individual trees. In our study, it was used to improve predictive accuracy and control over-fitting, which can be a problem in decision trees.
- Gradient-Boosted Decision Trees (GBDT): GBDT is another ensemble technique that builds the model in a stage-wise fashion. It constructs new trees that predict the residuals or errors of prior trees then combines these trees in a weighted manner to make the final prediction. In predicting IOP elevation, GBDT was particularly useful for handling various types of patient data and improving prediction accuracy over traditional decision trees.

Were employed to conduct the prediction of IOP elevation. For our data processing approach, each surgical procedure on an individual eye of a patient was treated as a separate sample. The dataset was divided into a training set and a testing set at a ratio of 9:1. Each model was then trained on the training set and tested on the testing set, with the test results reported. In this phase, we employed the grid search technique to find the most suitable hyperparameters for the current model, such as maximum depth and the number of estimators. All methods were experimented within the same framework to ensure fairness in comparison.

Furthermore, considering not all features of the samples might positively contribute to prediction accuracy due to the presence of redundant features in the patient characteristics, feature selection was performed before training the model. This process incorporated the clinical experience of senior doctors and the data analysis results from earlier in this study to filter out redundant feature information. Specifically, we initially identified 19 fixed important features based on the expertise of senior doctors (see Table 4, Fixed Features). These features were used as a foundational input for training and predicting in each model. Following this, based on the *p*-values from our hypothesis testing, we gradually increased the *p*-value threshold, which allowed for the introduction of additional features for experimentation (*p* < 0.1, 0.2, 0.3, 0.4, 0.5, and all features). The results of the prediction models on IOP elevation are shown in Table 4.

**Table 4:** Results of the prediction model on IOP elevation.

*p*-value	Model	Accuracy	Precision	Recall	F1-score
All (36 features)	DT	0.757	0.5	0.0769	0.1333
	LR	0.7664	0.5385	0.2692	0.359
	RF	0.7944	0.75	0.2308	0.3529
	GB	0.7757	0.5385	0.2692	0.359
Fixed features (19 features)	DT	0.757	0.5	0.0769	0.1333
	LR	0.7757	0.5833	0.2692	0.3684
	RF	0.785	0.6364	0.2692	0.3784
	GB	0.7757	0.5455	0.4615	0.5
*p* < 0.1 (28 features)	DT	0.757	0.5	0.0769	0.1333
	LR	0.7664	0.5385	0.2692	0.359
	RF	0.7944	0.6	0.4615	0.5217
	GB	0.785	0.5517	0.6154	0.5818
*p* < 0.2 (30 features)	DT	0.757	0.5	0.0769	0.1333
	LR	0.7664	0.5385	0.2692	0.359
	RF	0.7757	0.6	0.2308	0.3333
	GB	0.785	0.5517	0.6154	0.5818
*p* < 0.3 (31 features)	DT	0.757	0.5	0.0769	0.1333
	LR	0.7664	0.5385	0.2692	0.359
	RF	0.7664	1	0.0385	0.0741
	GB	0.785	0.5714	0.4615	0.5106
*p* < 0.4 (31 features)	DT	0.757	0.5	0.0769	0.1333
	LR	0.7664	0.5385	0.2692	0.359
	RF	0.7664	1	0.0385	0.0741
	GB	0.785	0.5714	0.4615	0.5106
*p* < 0.5 (32 features)	DT	0.757	0.5	0.0769	0.1333
	LR	0.7664	0.5385	0.2692	0.359
	RF	0.7757	0.5625	0.3462	0.4286
	GB	0.7944	0.5769	0.5769	0.5769

DT: decision tree, LR: Logistic Regression, RF: Random Forest, GB: Gradient Boosting.

**Figure 2:**
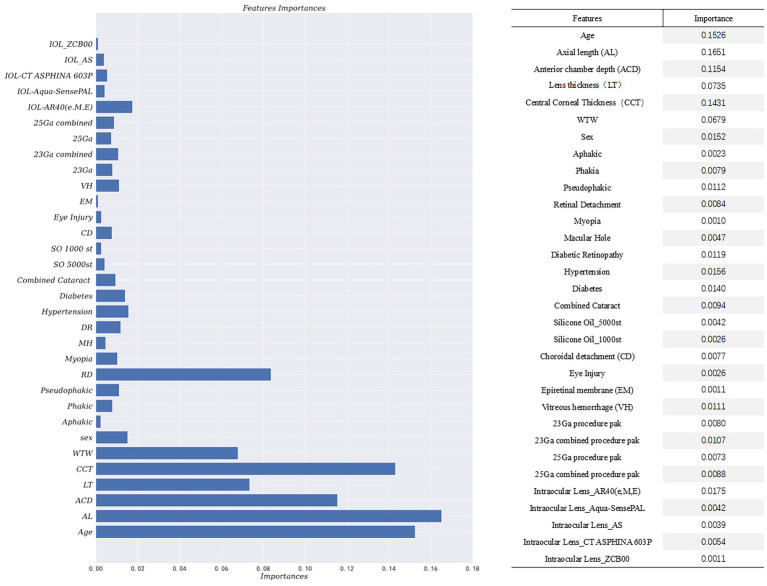
Feature importance provided by prediction model.

By incorporating the clinical expertise of senior doctors and considering features with *p*-values less than 0.5 from hypothesis testing, the Gradient-Boosted Decision Trees model achieved the highest accuracy of 0.7944. The feature importance provided by the prediction model was presented in Figure 2. The vertical axis represents the feature name, while the horizontal axis represents the corresponding feature importance value. It is worth noting that the sum of the importance of all features is equal to 1. The model assigns higher importance to certain features compared to others in the prediction process. The results of the prediction model indicate that factors such as age, biological parameters, and lens status play a more significant role. Specifically, the importance values for each feature are as follows: Age-0.1526, AL-0.1651, ACD-0.1154, LT-0.0735, CCT-0.1431, WTW-0.0679, Phakic-0.0079, Aphakia-0.0023, Pseudophakic-0.0112, Cata-ract-0.0094, RD-0.0084. These findings align with the results obtained from our single-factor analysis above. The code of prediction model can be found in supplementary materials.

## 4 Discussion

This study aimed to evaluate the risk factors and incidence of postoperative IOP elevation following vitreoretinal surgery with silicone oil tamponade in patients who have no prior history of glaucoma or ocular hypertension. Based on these findings, a predictive model was developed in the hope of early detection and treatment for these patients. The presence of preexisting glaucoma is widely recognized as a significant risk factor for IOP elevation following PPV and SO tamponade. Previous studies have reported an incidence of approximately 5.9% to 7% in patients who experienced elevated IOP after surgery and had a history of preoperative glaucoma or ocular hypertension (14, 15). In our study, we excluded patients with a history of glaucoma or ocular hypertension in order to identify additional risk factors. Elevated IOP commonly occurs within the first week after surgery in patients. Therefore, close monitoring and follow-up are essential during this period, along with timely initiation of appropriate treatment to achieve better treatment and prognosis.

Previous studies have considered diabetes, aphakic eye, and the viscosity of silicone oil as risk factors for IOP elevation after PPV combined with silicone oil tamponade (16, 17). In our study, analysis revealed that age, sex, hypertension, diabetes, myopia, retinal detachment, lens status and biological parameters were identified as risk factors for elevated IOP after PPV.

Our results indicated that younger patients are more prone to developing high IOP postoperatively. This finding is comparable with Pillai et al. (18), which suggested that the higher incidence of trabeculitis and anterior chamber inflammation in younger patients may contribute to the manifestation of high IOP symptoms following surgery due to their heightened inflammatory response. However, our study lacks clear evidence to support this conclusion, highlighting the need for future investigations.

Although several biological parameters, such as ACD, CCT, LT, and WTW distance, significantly influenced postoperative IOP in the present study, few prior studies have evaluated their effects on IOP elevation after vitreoretinal surgery with silicone oil filling. Previous studies have found a positive association between CCT and IOP values (19–21). Additionally, a tendency of higher IOP readings with longer axial length has been reported (22), while Hoffmann et al. (23) highlighted the high correlation of IOP measures with thicker central cornea, greater lens thickness, and longer posterior segment length. WTW distance has been found to be positively correlated with ACD. This suggested that WTW also has a positive association with IOP (24). However, these studies only reported the correlation between biological parameters and intraocular pressure, without analyzing the impact on the occurrence of elevated IOP after vitrectomy and silicone oil tamponade. In contrast, the present study is the first to investigate these relationships and found a high positive correlation between postoperative IOP elevation and AL, ACD, and WTW. Further exploration and explanation of the relationship between biological parameters and IOP is necessary.

Many articles have reported that aphakia is a strong risk factor for IOP elevation after PPV and SO tamponade (14, 25), and our study confirmed this finding. We also founded that patients with pseudophakia are also associated with an increased risk of IOP elevation. Chang (26) was the first to report that the diffusion of oxygen from the vitreous cavity into the anterior chamber is the main reason for elevated IOP in vitrectomized eyes. This diffusion caused alterations in the trabecular meshwork, resulting in reduced aqueous outflow, increased IOP, and the development of glaucoma (27, 28). Because the crystalline lens contains proteins that metabolize oxygen (29, 30), it has the potential to reduce oxidative stress on the trabecular meshwork and prevent oxidative damage. Additionally, it serves as a barrier, preventing oxygen from entering the anterior chamber. These factors could explain why our model estimates that lens status is a significant factor influencing postoperative IOP elevation.

Compared to previous studies (31–33), it has been shown that combined phacoemulsification and intraocular lens implantation (PE&IOL) have an additive effect on transient elevation of IOP, which was consistent with the findings of our prediction model. Furthermore, the long-term effect of cataract surgery on reducing IOP can result in reduced IOP during the late postoperative period. This could be attributed to the induction of greater inflammation in the trabecular meshwork by cataract surgery, resulting in increased IOP during the initial postoperative period. Additionally, the combination of PPV and cataract surgery can induce anterior segment inflammation and disrupt the blood-aqueous barrier (25, 34–36). The long-term reduction of IOP following cataract surgery can be attributed to improvements in outflow facility, deepening of the anterior chamber, and posteriorizing of the lens-iris diaphragm (37, 38).

Patients diagnosed with RD or VH showed a strong association with the development of elevated IOP in our study. Fujikawa et al. (39) reached a similar conclusion, indicating that the RD group had a significantly higher risk of post-vitrectomy IOP increase. Recent study findings (40) have suggested that the absence of an oxygen diffusion barrier in the vitreous leads to significantly elevated partial oxygen pressure within the vitreous cavity. In the case of patients diagnosed with RD and VH, we conducted a comprehensive vitrectomy, completely removing the peripheral vitreous, while performing a core vitrectomy for other patients. This procedure could lead to increased oxygen stress on the trabecular meshwork in the eyes of RD patients (41), potentially resulting in elevated IOP.

Elevated IOP is a common and severe complication of PPV with silicone oil tamponade. Clinicians typically manage high postoperative IOP through the administration of IOP-lowering eye drops or early removal of intraocular silicone oil to avoid optic nerve damage, but the latter approach may induce secondary complications like recurrent retinal detachment. Thus, it is essential from a clinical perspective to examine the potential risk factors associated with postoperative hypertension and develop corresponding interventions to mitigate its occurrence. Furthermore, given the high incidence of myopia and cataract in China, our study introduces biological parameters as factors for a comprehensive systemic analysis. Leveraging a larger data set, our study provides a more thorough and precise analysis of the potential risk factors linked to postoperative high IOP. Moreover, our statistical findings and prediction model lay the foundation for future prospective studies.

## 5 Conclusion

In conclusion, IOP elevation is a common complication following PPV with SO tamponade. Our study identified several risk factors, including age, sex, hypertension, diabetes, myopia, retinal detachment, lens status and biological parameter. Additionally, we conducted prediction model using GBDT to predict IOP elevation, achieving an accuracy of 0.7944. Knowledge of incidence, risk factors, and mechanism of IOP rise following PPV is essential for the postoperative follow-up and management of patients.

## Data availability statement

The raw data supporting the conclusions of this article will be made available by the authors, without undue reservation.

## Ethics statement

The studies involving humans were approved by the ethics committee of The First Affiliated Hospital of Nanjing Medical University, 2022-SR-200. Exception to the requirement of informed consent. The studies were conducted in accordance with the local legislation and institutional requirements. Written informed consent for participation was not required from the participants or the participants’ legal guardians/next of kin in accordance with the national legislation and institutional requirements.

## Author contributions

WF: Conceptualization, Funding acquisition, Methodology, Resources, Writing – review & editing, Project administration. CZ: Formal analysis, Investigation, Methodology, Software, Writing – original draft, Conceptualization, Validation, Visualization. LG: Data curation, Formal analysis, Methodology, Writing – original draft, Conceptualization, Validation, Visualization. NS: Data curation, Investigation, Writing – review & editing. JC: Data curation, Writing – review & editing. SS: Software, Writing – review & editing. YW: Conceptualization, Resources, Supervision, Validation, Visualization, Writing – review & editing. SY: Conceptualization, Funding acquisition, Resources, Supervision, Validation, Visualization, Writing – review & editing.
